# Supplementary material for: Adherence to Annual Fundus Exams among Chinese Population with Diagnosed Diabetes
Source: J Clin Med. 2022 Nov 21;11(22):6859. doi: 10.3390/jcm11226859 (PMC9697630; doi:10.3390/jcm11226859)
Supplement: Supplementary file 1 [file jcm-11-06859-s001.zip › jcm-1987829-supplementary.pdf]

**Table S1.** Adherence rate of diabetic patients complying with annual fundus exam.

| Annual Fundus Exam (%) | 2011 ($n$ = 985) | 2013 ($n$ = 1025) | 2015 ($n$ = 1027) | 2018 ($n$ = 1050) | $p < 0.001$ |
|---|---|---|---|---|---|
| Yes | 231 (23.5 %) | 157 (15.3 %) | 180 (17.5 %) | 226 (21.5 %) | |
| No | 754 (76.5 %) | 868 (84.7 %) | 847 (82.5 %) | 824 (78.5 %) | |

Adherence rates (proportion) to annual fundus examinations among diabetic patients in China. Changes in adherence rates over 7 years were assessed by Pearson$\chi$2.

**Table S2.** Changes in descriptive characteristics of Non-adherent sample from 2011 to 2018.

| Variables | 2011 ($n$ = 985) | 2013 ($n$ = 1025) | 2015 ($n$ = 1027) | 2018 ($n$ = 1050) | $p$ Value |
|---|---|---|---|---|---|
| Gender | | | | | 0.9563 |
| Male | 326 (43.2 %) | 381 (43.9 %) | 378 (44.6 %) | 361 (43.8 %) | |
| Female | 428 (56.8 %) | 487 (56.1 %) | 469 (55.4 %) | 463 (56.2 %) | |
| Age | 61.07± 9.24 | 61.98± 9.27 | 63.68± 9.10 | 62.61± 9.39 | <0.001 |
| Education | | | | | 0.9255 |
| Illiterate | 197 (26.1 %) | 207 (23.8 %) | 208 (24.6 %) | 219 (26.6 %) | |
| Less than elementary school | 304 (40.3 %) | 371 (42.7 %) | 356 (42.0 %) | 348 (42.2 %) | |
| Middle school | 146 (19.4 %) | 168 (19.4 %) | 166 (19.6 %) | 150 (18.2 %) | |
| High school or vocational school | 84 (11.1 %) | 96 (11.1 %) | 87 (10.3 %) | 89 (10.8 %) | |
| college and above | 23 (3.1 %) | 26 (3.0 %) | 30 (3.5 %) | 18 (2.2 %) | |
| Marital status | | | | | 0.3206 |
| Yes | 611 (81.0 %) | 723 (83.3 %) | 699 (82.5 %) | 660 (80.1 %) | |
| No | 143 (19.0 %) | 145 (16.7 %) | 148 (17.5 %) | 164 (19.9 %) | |
| Smoke | | | | | 0.0162 |
| Yes | 263 (34.9 %) | 327 (37.7 %) | 354 (41.8 %) | 292 (35.4 %) | |
| No | 490 (65.1 %) | 541 (62.3 %) | 493 (58.2 %) | 532 (64.6 %) | |
| Drinking status | | | | | 0.7025 |
| Drink more than once a month | 130 (17.3 %) | 162 (18.7 %) | 162 (19.1 %) | 171 (20.8 %) | |
| Drink but less than once a month | 50 (6.6 %) | 64 (7.4 %) | 58 (6.8 %) | 59 (7.2 %) | |
| None of these | 573 (76.1 %) | 642 (74.0 %) | 627 (74.0 %) | 594 (72.1 %) | |
| Using diabetes medication | | | | | <0.001 |
| Yes | 512 (67.9 %) | 534 (61.5 %) | 512 (60.4 %) | 469 (56.9 %) | |
| No | 242 (32.1 %) | 334 (38.5 %) | 335 (39.6 %) | 355 (43.1 %) | |
| Non-medication treatments | | | | | 0.0749 |
| Yes | 298 (39.5 %) | 335 (38.6 %) | 298 (35.2 %) | 340 (41.3 %) | |
| No | 456 (60.5 %) | 533 (61.4 %) | 549 (64.8 %) | 484 (58.7 %) | |
| Multi-morbidities | | | | | <0.001 |
| Yes | 651 (86.3 %) | 738 (85.0 %) | 753 (88.9 %) | 655 (79.5 %) | |
| No | 103 (13.7 %) | 130 (15.0 %) | 94 (11.1 %) | 169 (20.5 %) | |
| Vision impairment | | | | | <0.001 |
| Yes | 382 (50.7 %) | 428 (49.3 %) | 344 (40.6 %) | 296 (35.9 %) | |
| No | 372 (49.3 %) | 440 (50.7 %) | 503 (59.4 %) | 528 (64.1 %) | |
| Regular physical exam | | | | | <0.001 |
| Yes | 496 (65.8 %) | 479 (55.2 %) | 449 (53.0 %) | 458 (55.6 %) | |
| No | 258 (34.2 %) | 389 (44.8 %) | 398 (47.0 %) | 366 (44.4 %) | |
| Insurance cover | | | | | <0.001 |
| Yes | 709 (94.0 %) | 820 (94.5 %) | 816 (96.3 %) | 806 (97.8 %) | |
| No | 45 (6.0 %) | 48 (5.5 %) | 31 (3.7 %) | 18 (2.2 %) | |

**Table S3.** Different sample size of diabetes in CHARLS 2011 and adherence rate to annual fundus exam based on four definitions of diabetes.

| Year | Self-Reported Diabetes | Reference Defined Diabetes | Unaware of Diabetes | Total Diabetes |
|---|---|---|---|---|
| 2011 | 985 | 1544 | 1157 | 2142 |
| Annual fundus exam | | | | |
| Yes | 233 | 89 | 0 | 233 |
| No | 754 | 1455 | 1157 | 1909 |
| Adherence rate | 23.65% | 5.76% | None | 10.88% |

Reference defined diabetes: Fasting blood glucose (FBG), random blood glucose (RBG) or HbA1c measurement (FBG ≥ 126 mg/dl, RBG ≥ 198 mg/dl or HbA1c ≥ 6.5%); Unaware af diabetes: Abnormal blood test but responded 'no diabetes' in questionnaire; Total diabetes: Self-reported diabetes or defined by blood test.
